# Is an EAA > 0.6 in severe COVID-19 patients synonymous with a toxic and pro-inflammatory endotoxin profile, and should we treat it?

**DOI:** 10.1186/s40635-021-00376-w

**Published:** 2021-03-12

**Authors:** Patrick M. Honore, Sebastien Redant, David De Bels

**Affiliations:** ICU Department, Centre Hospitalier Universitaire Brugmann, Brugmann University Hospital, Place Van Gehuchtenplein, 4, 1020 Brussels, Belgium

## Introduction

In a recent issue of Intensive Care Medicine Experimental, Sirivongrangson et al. reported the findings of their study examining endotoxemia in patients with severe COVID-19 [1]. Of the 19 patients studied, approximately half displayed elevated blood endotoxin levels, measured by the endotoxin activity assay (EAA), or increased intestinal permeability, reflected by increased serum levels of (1–3)-β-D-Glucan (BG), with a third of patients having both. Although only one patient had a positive blood culture, 18 patients were positive for 16S rRNA gene amplification. These findings raise the question of what role bacterial products may have in the pathogenesis of COVID-19.

## Is there a link between obesity and COVID-19 severity via endotoxin?

The study by Sirivongrangson et al. has demonstrated the presence of endotoxin with an EAA > 0.6 in blood of patients with COVID-19 acute respiratory distress syndrome (CARDS) [1]. The EAA and BG levels detected in this cohort of patients with CARDS were comparable to those reported in the authors’ previous study in 136 patients with severe sepsis or septic shock [1]. Recent insights suggest that obese patients are more at risk for COVID-19, as well as associated hospitalization, intensive care unit (ICU) admission, and mortality [2]. Unfortunately, the study by Sirivongrangson et al. does not provide information on the BMI of included patients [1]. It is possible that endotoxin originated from secondary bacterial infections (all caused by Gram-negative bacteria) which occurred in several patients within the timeframe of the EAA measurements [1]. An alternative hypothesis is that the endotoxin originated from the gut microbiota. The structure of lipid A is diverse among bacterial species and the number of acyl chains determines its immunostimulatory potential and thus toxicity [2]. In the gut of every human, more than 10^14^ microorganisms (bacteria, archaea, viruses, and eukaryotic microbes) colonize the gastrointestinal (GI) tract and are commonly known by the term ‘gut microbiome’ [2]. The human gut microbiota is mostly composed of two dominant microorganisms (representing more than 90% of the total community)—*Bacteroidetes* (Gram-negative bacteria) and *Firmicutes* (mostly Gram-positive bacteria) [2]. *Bacteroidetes* contribute to 79% of the endotoxin biosynthesis in healthy volunteers and 92.4% in the human microbiome [2]. Under normal conditions, the total endotoxin produced in the healthy adult human gut is immunosilent/immunoinhibitory, meaning that it has a very limited capacity to activate the toll-like receptor 4 (TLR4)-NF-κB pathway and elicit the production of inflammatory cytokines [2]. Furthermore, under-acylated lipid A structures across the order *Bacteroidales* are probably the main reason for this immunosilent endotoxin [2]. Individuals with obesity have a significantly higher level of *Firmicutes* and lower level of *Bacteroidetes* [2]. This could induce a change in the toxicity of endotoxin from the gut. Indeed, due to gut microbiome dysbiosis, intestinal endotoxin composition in individuals with obesity could be shifted away from immunosilent/immunoinhibitory *Bacteroidetes* endotoxin subtypes in favor of various proinflammatory endotoxin subtypes (phyla producing more inflammatory endotoxin) [2]. Recently, a close link has been described between obesity-associated gut microbiome dysbiosis and derangements in the regulation of gut permeability [2]. Thus, endotoxins could move into the circulatory system through direct diffusion due to intestinal paracellular permeability resulting in endotoxemia [2]. Subsequently, lipid A initiates a signaling cascade resulting in activation of various proinflammatory pathways and increases oxidative stress upon binding to TLR4 [2]. It is possible that obese patients with severe COVID-19 not only possess more toxic and more pro-inflammatory endotoxin in their GI tract, but also have higher intestinal permeability resulting in increased transfer of endotoxin into the bloodstream. In the study by Sirivongrangson et al., elevated serum (1,3)-β-D-Glucan (BG) levels indicated that intestinal barrier permeability was increased [1]. However, given that Proteobacteria was the major phylum detected, a shift towards a more toxic and inflammatory endotoxin is less likely.

## If endotoxin does play a role in the pathophysiology and severity of COVID ARDS, what can we do?

If we look carefully at the data provided in the study by Sirivongrangson et al., patients with a high EAA tended to require more mechanical ventilatory support than the low EAA group (62.5 vs 45.5%) [1]. The proportion of patients requiring vasopressors, prone position, and extracorporeal membrane oxygenation (ECMO) did not differ between EAA groups [1]. No patients died during the 28 days following enrollment [1]. The fact that the groups with and without EAA > 0.6 had no difference in the need for vasopressors or ECMO, in addition to Proteobacteria being the predominant phylum detected, supports the hypothesis that there was no shift towards a more toxic and proinflammatory endotoxin. On the other hand, the fact that patients with an EAA > 0.6 required more ventilatory support may perhaps justify the use of polymyxin B (PMx) to remove endotoxin [3]. Of note, this study was performed in March/April 2020 before we the data on the effects of dexamethasone from the RECOVERY trial became available [4]. Lastly, as previously noted, we do not know whether (some of) the patients of Sirivongrangson et al. were obese or not. This would be interesting information; if the potential shift to a more toxic and pro-inflammatory endotoxin in obese patients is taken into account, the presence of an EAA > 0.6 in obese patients with severe COVID-19 might justify the use of PMx, in cases of shock. 

## Conclusions

The presence of an EAA > 0.6 does not necessarily indicate the presence of toxic or proinflammatory endotoxin in the circulation. A shift in the gut microbiota is required to produce a more toxic and inflammatory endotoxin, in the case of obese patients with COVID-19, and it is not clear that that is the case in this study. The potential role of PMx in the treatment of obese patients with severe COVID-19 and a shift to proinflammatory endotoxin warrants further study [5].

## Data Availability

Not applicable.

## Abbreviations

EAA: Endotoxin activity assay
CARDS: COVID-19 acute respiratory distress syndrome
ARDS: Acute respiratory distress syndrome
ICU: Intensive care unit
GI: Gastrointestinal
TLR4: Toll-like receptor 4
NF-κB: Nuclear factor-κB
BG: (1,3)-β-D-glucan
ECMO: Extracorporeal membrane oxygenation
PMx: Polymyxin B
